# Tat-Based Therapies as an Adjuvant for an HIV-1 Functional Cure

**DOI:** 10.3390/v12040415

**Published:** 2020-04-08

**Authors:** Hongping Jin, Dongsheng Li, Min-Hsuan Lin, Li Li, David Harrich

**Affiliations:** 1 Department of Cell and Molecular Biology, QIMR Berghofer Medical Research Institute, Herston, QLD 4006, Australia; Hongping.Jin@qimrberghofer.edu.au (H.J.); Dongsheng.Li@qimrberghofer.edu.au (D.L.); Min-Hsuan.Lin@qimrberghofer.edu.au (M.-H.L.); 2 Australian Institute for Bioengineering and Nanotechnology, The University of Queensland, St Lucia, QLD 4072, Australia; l.li2@uq.edu.au

**Keywords:** Tat, HIV transcription, Nullbasic, didehydro-cortistatin A, block and lock, HIV functional cure, Tat vaccine, triptolide

## Abstract

The human immunodeficiency virus type 1 (HIV) establishes a chronic infection that can be well controlled, but not cured, by combined antiretroviral therapy (cART). Interventions have been explored to accomplish a functional cure, meaning that a patient remains infected but HIV is undetectable in the blood, with the aim of allowing patients to live without cART. Tat, the viral transactivator of transcription protein, plays a critical role in controlling HIV transcription, latency, and viral rebound following the interruption of cART treatment. Therefore, a logical approach for controlling HIV would be to block Tat. Tackling Tat with inhibitors has been a difficult task, but some recent discoveries hold promise. Two anti-HIV proteins, Nullbasic (a mutant of Tat) and HT1 (a fusion of HEXIM1 and Tat functional domains) inhibit viral transcription by interfering with the interaction of Tat and cellular factors. Two small molecules, didehydro-cortistatin A (dCA) and triptolide, inhibit Tat by different mechanisms: dCA through direct binding and triptolide through enhanced proteasomal degradation. Finally, two Tat-based vaccines under development elicit Tat-neutralizing antibodies. These vaccines have increased the levels of CD4^+^ cells and reduced viral loads in HIV-infected people, suggesting that the new vaccines are therapeutic. This review summarizes recent developments of anti-Tat agents and how they could contribute to a functional cure for HIV.

## 1. Introduction

The human immunodeficiency virus type 1 (HIV) was discovered in 1983 and is the cause of acquired immunodeficiency syndrome (AIDS). Antiretroviral therapy using two or more drugs, cART, can effectively control but not cure HIV infection. Most cART drugs inhibit one of the viral enzymes, reverse transcriptase, integrase or protease, although HIV capsid and the cellular receptors for HIV have also been successfully targeted to inhibit virus replication. cART suppresses viremia to undetectable levels, however, for most people living with HIV (HIV^+^ people), the infection is lifelong. This is because HIV persists in long-lived cell reservoirs in a wide variety of tissues [1,2,3], mainly in resting memory CD4^+^ T cells [4,5,6,7,8]. Recent evidence shows that macrophages in brain tissue and urethral mucosa are also HIV reservoirs [9,10], and that these reservoirs are difficult to treat with cART. Ongoing low levels of viral replication and/or homeostatic proliferation of latently infected cells are two proposed explanations for chronic HIV infection [11,12]. The cART regimens currently available do not inhibit viral transcription in HIV-infected cells. Therefore, when cART is discontinued or ineffective, viral rebound in HIV^+^ people is a reasonable certainty [13].

The precise mechanisms that establish HIV latency are not fully understood. Several factors impacting transcriptional silencing have been reviewed in detail elsewhere, including the site of HIV integration in host genome, the availability of host transcription factors including NF-κB and cyclin T1 (CycT1), and chromatin structure [14,15,16,17,18]. Dynamic interplay between the activation state of T cells infected by HIV and viral regulatory proteins [19,20,21], especially Tat [22,23,24], is an important regulator of HIV latency and reactivation. When CD4^+^ T cells undergo an effector-to-memory T cell transition, there can be a temporary upregulation in expression of CCR5, a viral co-receptor that along with CD4 facilitates the entry of HIV into the cell, and a rapid downregulation of cellular gene expression. HIV infection of these cells is characterized by suppressed HIV gene transcription, which establishes latency [25]. Cell models show polarized effector T cells infected by HIV enter into a quiescent stage, forcing HIV into latency [26]. In a study of populations of newly infected primary T cells, an analysis of viral RNA by single cell RNAseq indicated that the transcriptional programs of T cells with markers of central memory and naïve T cells are associated with viral latency [27], whereas Tat has been implicated as a master controller of HIV latency regardless of the cell state [22,23,24]. Another recent study demonstrated that HIV silencing and reactivation are heterogeneous and are affected by factors intrinsic to the virus. By using naturally occurring genetic variations that affect Tat expression, this study showed that viruses that poorly express Tat require a proportionally increased stimulus to achieve reactivation from latency. This result suggests that silencing and reactivation behaviors of HIV exist in a spectrum and are influenced by factors intrinsic to the virus, including Tat [28].

As Tat is essential for virus transcription and replication, antiviral agents that block Tat function might block virus production by infected cells. Here we provide a review of Tat function in HIV transcription, recent discoveries and developments of anti-Tat agents, and the possible contribution of anti-Tat agents to a functional cure for HIV infection.

## 2. Tat Transactivation of HIV Transcription

**Figure 1:**
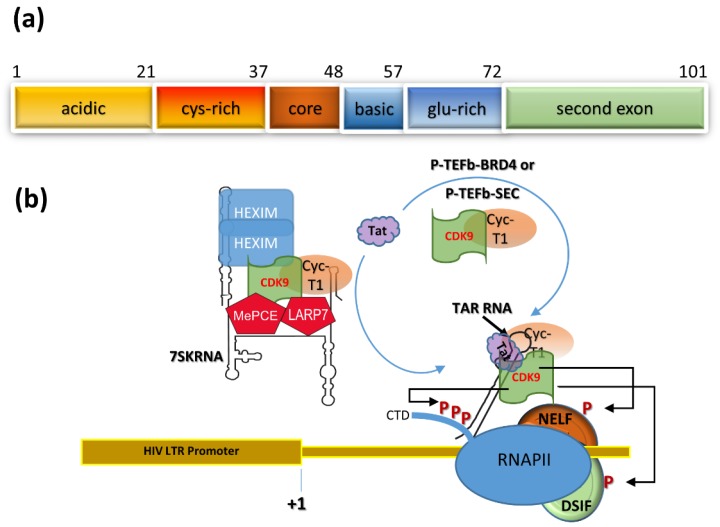
(**a**) A schematic of Tat domains including acidic, cysteine (cys)-rich, core, basic (also called arginine rich), glutamine (glu)-rich and the second exon. (**b**) A model of transactivation where Tat recruits P-TEFb from the 7SK snRNP complex, where 7SK-RNA, LARP7, and MePCE make the core 7SK snRNP, which can bind to and inhibit CDK9 activity of P-TEFb by HEXIM. Tat recruits P-TEFb from sources that include the 7SK snRNP, P-TEFb-BRD4, and P-TEFb-SEC complexes. The Tat:P-TEFb complex binds to TAR RNA, where CDK9 phosphorylates NELF (leads to its release) and DSIF, as well as hyperphosphorylating the C-terminal domain (CTD) of RNAP II, which then undertakes efficient synthesis and elongation of full length viral mRNA transcripts.

Tat is an essential viral protein for HIV replication. The functions of Tat, including its roles in HIV transcription, have been described in detail elsewhere [29]. Briefly, Tat is a small viral protein comprising 101 amino acids, in most HIV strains, encoded by two exons (Figure 1a) [30]. The first exon encodes 72 amino acids and is divided into 5 regions: a proline-rich acidic N-terminus (amino acids 1–21), a cysteine-rich region (amino acids 22–37), a hydrophobic core region (amino acids 38–48), an arginine-rich basic region (amino acids 49–57), and a glutamine-rich region (amino acids 58–72). Exon 2 encodes a C-terminal domain. The first 48 amino acids are the activation domain, followed by the basic domain (amino acids 49–57). The basic domain of Tat is required for Tat’s nuclear localization and the binding of Tat to the HIV RNA stem loop structure, called the transactivation response (TAR) RNA, as well as many other Tat functions [31]. Without Tat, HIV transcription by RNA polymerase II (RNAP II) is very inefficient and viral RNA transcripts corresponding to TAR RNA are less than 60 nucleotides long [32,33,34,35], as observed in cell line models of HIV latency and in HIV^+^ people [36]. Importantly, non-productively infected cells can be activated by cell stimuli to make infectious viruses [36].

A key point is that the HIV promoter is capable of a low basal level of transcription [37], that may stochastically produce a full length viral mRNA [22,38], which can be spliced into *tat*-encoding mRNA. The availability of Tat in an HIV-infected cell initiates a powerful feedback system that greatly increases HIV transcription (reviewed in [29,39]). Briefly, the activation domain of Tat can bind to the positive transcription elongation factor b (P-TEFb), a cellular complex composed of cyclin T1 (CycT1) and CDK9. In the cell, P-TEFb can be associated with the 7SK small nuclear ribonucleoprotein (snRNP), the Brd4 complex or the super elongation complex (SEC), and Tat can exploit P-TEFb from each of these resources [40]. Tat mediates binding of the Tat:P-TEFb complex at the HIV long terminal repeat (LTR) promoter (Figure 1b), which is achieved by specific binding of Tat:P-TEFb to TAR RNA. This leads to several important events that upregulate HIV transcription up to 100-fold over basal expression levels: 1) CDK9 phosphorylates the RNAP II associated transcriptional suppressors DRB sensitivity-inducing factor (DISF) and the negative transcription elongation factor (NELF), which dissociates from the RNAP II elongation complex; 2) CDK9 hyperphosphorylates the C-terminal repeat domain of RNAP II, which greatly increases productive transcription of full length viral mRNA, and; 3) cellular and viral proteins including Tat recruit chromosome remodeling histone acetyltransferases, so that nucleosomes flanking the LTR promoter are in an open euchromatin configuration of highly transcribed genes in cells.

## 3. A Tat-Based Block-and-Lock Functional Cure Strategy

Recently, a functional cure for HIV has been proposed that uses a block-and-lock strategy, in which anti-HIV agents that prevent transcription of HIV lock the cellular HIV reservoir into a “deep”-latent, transcriptionally silent state, preventing rebound after cessation of cART [41,42,43,44,45,46,47,48,49,50]. The block-and-lock strategy differs from the shock-and-kill strategy [51,52], where latency-reversing agents (LRAs) are used to stimulate and reactivate, or shock, latent HIV in the cellular reservoirs so that infected cells die as a direct result of virus production or are killed by the immune response to HIV. As Tat transactivation is a critical step in HIV transcription, agents that block Tat will inhibit HIV replication and viral transcription. Recent modeling suggests inhibitors that interact with Tat and disrupt the transactivation cycle, leading to epigenetic modifications of nucleosomes in the viral promoter, could cause prolonged inactivation, or deep latency, of HIV transcription [53]. The identification of Tat inhibitors is the result of more than two decades of research [54]. While none of the transactivation inhibitors discovered has advanced to clinical use, research on several Tat targeting agents continues.

## 4. Tat Trans-Dominant Negative Proteins and Nullbasic

### 4.1. Tat Trans-Dominant Negative Proteins

Tat mutants that inhibit HIV transactivation were first reported more than 30 years ago and were referred to as trans-dominant negative (TDN) proteins [55,56]. Early studies used a 72 amino acid TDN Tat encoded by the first exon where some but not all basic domain residues were mutated to glycine; this mutant Tat was found to inhibit HIV transcription if present at ~20-fold excess compared to wild type Tat. While the first TDN Tats were not powerful inhibitors of HIV transcription, a combined treatment of TDN Tat and TDN Rev (called M10) [57,58,59] showed greater inhibition of HIV replication than either as an individual treatment [60].

The basic domain of Tat is essential for TAR RNA interaction and has versatile roles in the interaction of Tat and the host cell factors, which have been reviewed by Kurnaeva et al. [31]. Tat interacts with HIV reverse transcriptase and stimulates reverse transcription [61,62]. Acetylation of Lys50 and Lys51 in the basic domain appears to regulate this activity [63]. Nullbasic is a 101 amino acid TDN Tat in which the entire basic domain is replaced by Gly and Ala residues [64]. Studies show that, when expressed in cell lines, Nullbasic inhibits Tat transactivation [64], reverse transcription [64,65] and Rev activity [64,66,67] (Table 1).

### 4.2. Nullbasic Inhibits HIV Transcription and Reactivation from Latency

When Jurkat cells expressing Nullbasic were infected with HIV, HIV replication was completely inhibited [69]. Although HIV proviral DNA could be detected in chromosomes of the infected cells, no measurable HIV RNA was identified in these cells. A co-culture experiment where infected Jurkat cells were mixed with uninfected Jurkat cells failed to rescue infectious virus, suggesting that HIV production was blocked in the infected cells. Transcription from proviral DNA in the cells could not be reactivated by LRAs including phorbol ester 12-myristate-13-acetate (PMA), which activates protein kinase C and reactivates NF-κB [88], and JQ-1, which binds to BRD4 and enables increased Tat binding to P-TEFb [89]. The histone deacetylase inhibitor suberoylanilide hydroxamic acid (SAHA), which is a strong activator of latent cells [90,91,92,93], only modestly stimulated HIV transcription [69]. This suggests that Nullbasic forces HIV into a latent state in Jurkat cells, refractory to LRA reactivation.

Nullbasic also strongly inhibited HIV replication in human primary CD4^+^ cells [68,70], and against different HIV subtypes [94]. This is most likely because Nullbasic targets cellular factors such as P-TEFb [66] that are essential for replication by all HIV subtypes. Nullbasic was tested in two mouse models of acute HIV infection where mice were engrafted with primary human CD4^+^ cells. The cells were either transduced with Nullbasic first and then infected with HIV (pre-infection treatment), or infected with HIV first and then transduced with Nullbasic (post-infection treatment) [70]. In the pre-infection model, Nullbasic inhibited HIV transcription up to 2800 fold in CD4^+^ cells recovered from organs, and no HIV was detected in blood samples [70]. In the post-infection model, the inhibitory effect of Nullbasic was less robust, but suggested that Nullbasic provided CD4^+^ cells a survival benefit [70].

A gene therapeutic approach to cure HIV remains an important area of research because of the potential for durable antiviral effects. Nullbasic inhibits three different steps of HIV replication, which may explain, in part, why it is a potent inhibitor of HIV. Given the inhibition observed in this simple animal model of an acute and active HIV infection, Nullbasic could be a part of a block-and-lock functional cure strategy as a gene therapy candidate.

## 5. A HEXIM1-Tat Fusion Protein that Inhibits HIV Replication

P-TEFb activity is controlled by the 7SK snRNA complex [95]. In this complex, HEXIM1 binds to the 7SK snRNA through its arginine-rich RNA-binding domain (ARM) [96] and inhibits the kinase activity of CDK9 in P-TEFb complex with its central inhibitory domain (ID) [97,98]. HEXIM1 and its paralog HEXIM2 inhibits Tat activity by recruiting CycT1 to 7SK snRNA [71]. A fusion protein named HT1 was derived from HEXIM1 (amino acids 150–220, including ARM and ID domains) and Tat (amino acids 1–48; the domain binds to P-TEFb) [72,99] (Table 1). Cell based experiments demonstrated that HT1 inhibited HIV transcription by binding to TAR RNA, inactivated CDK9 activity of P-TEFb and competed with Tat for binding to P-TEFb. Expression of HT1 had no significant effect on host cell gene expression and growth. In cell line models of HIV latency, HT1 inhibited viral reactivation by PMA stimulation [72]. These results support the investigation of HT1 as a potential gene therapy agent in a block-and-lock functional cure strategy. However, HT1 has not been evaluated in animal models of HIV infection.

## 6. Two Compounds that Inhibit Tat Activity

### 6.1. Didehydro-Cortistatin A (dCA)

dCA is a synthetic analogue of the natural steroidal alkaloid Cortistatin A (CA) from the marine sponge *Corticium simplex* (Table 1) [100]. CA was reported to bind and inhibit mediator kinases including CDK8, CDK11, and CDK19 [101,102], but does not inhibit HIV transcription [103]. dCA potently inhibited Tat transactivation by specifically binding to Tat via the basic domain [73], but did not bind to CDK9 in the P-TEFb complex. dCA also specifically bound to the basic domain of HIV-2 Tat [73] and SIV Tat [76]. The binding of dCA to the basic domain of Tat occurred across HIV subtypes A to E in the main group M [104], but dCA did not bind to the basic domain of Rev and HEXIM1 [74]. dCA caused a redistribution of Tat in the nucleus from the nucleolus to the nucleoplasm and periphery of the nucleolus in a dose-dependent manner [73]. In vitro cell based experiments have demonstrated that dCA inhibited virus transactivation by Tat in different HIV subtypes [104] and SIV [76]. dCA did not inhibit the initiation of transcription but inhibited the interaction of RNAP II with the LTR promoter [46].

Histone modifications regulate the accessibility of chromatin DNA to transcription factors, and acetylation of histone proteins in nucleosomes is required for HIV transactivation [105]. Research using the latently infected OM-10.1 cell line showed that the inhibition of Tat by dCA resulted in an extremely repressive chromatin environment at the HIV promoter, characterized by decreased H3K27 acetylation levels at nucleosome 1 (located downstream of the HIV LTR transcription start site) and limited PBAF recruitment [75]. PBAF is a type of SWI/SNF chromatin remodeler recruited by acetylated Tat to the viral promoter [106]. The activity of dCA is directly correlated to the strength of the Tat-TAR feedback loop [75]. dCA only partially blocked HIV reactivation in U1 cells and produced no significant inhibition of HIV reactivation of ACH2 cells [75]. This may be because these cell lines harbor defective HIV proviruses that have either a defect in TAR RNA (U1 cells) or Tat (ACH2 cells) that incompletely impede transactivation, so that increased basal levels of transcription in these cell lines can be induced by activation of NF-κB. dCA prevents HIV reactivation from latency in primary CD4^+^ cells derived from infected individuals and in many cellular models of latency [46]. In a bone marrow-liver-thymus (BLT) HIV mouse model, adding dCA to ART reduced viral mRNA in tissues, and both delayed and reduced HIV rebound after an ART interruption [107]. These results suggest that dCA may be useful clinically.

HIV can rapidly become resistant to any single anti-viral drug, so it is not surprising that HIV strains resistant to dCA emerged from long-term cultures [108,109]. No mutations in Tat and TAR were identified in these strains. The mutations in the dCA resistant strains were identified in the LTR region, Nef and Vpr. Mutations in the LTR region increased basal HIV transcription by 10- to 30-fold compared the wild type LTR, while the Nef and Vpr mutants increased NF-κB nuclear translocation, thereby facilitating transcription from the HIV LTR promoter. In primary CD4^+^ cells, dCA resistant virus produced up to 150-fold more virus than WT in the presence of dCA. Whether dCA-resistant strains can sustain infection in vivo remains an important question.

Clearly dCA is a promising anti-Tat agent that will likely be tested in primate models of HIV infection. It remains to be seen if dCA will be a viable addition to traditional ART as part of a block-and-lock HIV cure strategy.

### 6.2. Triptolide

Triptolide is a natural product extracted from a traditional Chinese herbal medicine, *Tripterygium wilfordii* Hook F, which has been used for the treatment of rheumatoid arthritis [110,111]. Triptolide also exhibits anti-inflammatory and anti-cancer properties [112,113,114]. The anti-inflammatory mechanism involves inhibition of NF-κB activation and DNA binding of NF-AT to enhancer sites in the IL-2 promoter [115]. Triptolide is a global transcriptional inhibitor of RNAP I, II and III [116,117,118,119], which partly explains its anti-cancer properties. Triptolide inhibits both total RNA and mRNA de novo synthesis in A549 cells. Triptolide represses the expression of RPB1 (the main RNAP II subunit) and MYC proteins, inducing an accumulation of p53 in A549 cells [116]. Triptolide also binds covalently to XPB, a subunit of TFIIH (a component of the pre-initiation complex of RNAP II), and blocks transcriptional initiation [120]. Triptolide can alter RNAP II promoter occupancy [121]. A recent paper showed that triptolide reduced the formation of TFIIIB, a multi-subunit transcription factor for RNAP III at the promoters of tRNAs and 5S rRNA in colorectal cancer cells [119].

Triptolide has also been investigated as an anti-HIV reagent at concentrations that do not affect global cellular transcription [77] (Table 1). The anti-HIV activity of Triptolide has been tested in HeLa-derived TZM-bl cells [122,123,124], which have a Tat-responsive HIV LTR promoter expressing reporter genes encoding luciferase and beta-lactamase, as well as in Jurkat cells and human peripheral blood cells infected with different HIV strains (HIV_NL 4-3_, HIV_LAI_ and HIV_BaL_). Triptolide inhibited HIV replication from about 89% to >99% in these cells. While triptolide did not affect the viability of TZM-bl and Jurkat cells, it reduced the viability of primary blood cells by about 25%. Time-of-addition experiments indicated that triptolide inhibited HIV transcription by targeting Tat. Triptolide was reported to reduce Tat steady-state levels by enhancing its proteasomal degradation, which could be blocked by the proteasomal inhibitor MG132. Protein arginine methyltransferase 6 (PRMT6) can methylate Tat, Rev and nucleocapsid protein and regulate Tat nuclear localization. Research has suggested that overexpression of PRMT6 can increase Tat steady-state levels in most cell lines [125]. A connection between PRMT6 and triptolide effects on Tat steady-state levels has not been investigated, but analysis of Tat mutants suggested an interaction between Tat with PRMT6 and the triptolide-mediated decrease of Tat steady-state levels both required the Tat activation domain.

However, due to its global inhibition of transcription, the clinical application of triptolide has been limited by its toxicity and side effects [126]. The utility of triptolide and *Triptolide wilfordii* as alternative ART await outcomes form clinical trials (NCT02219672 and NCT03403569).

## 7. Anti-Tat Vaccines that may Compliment cART

The prospect that a therapeutic anti-Tat vaccine could be an important approach to tackle HIV infection has been long argued [127]. As a powerful activator of HIV transcription, Tat enables rapid viremia and is hypothesized to regulate establishment of a cellular reservoir of infected cells [22,128]. The high mutability of HIV and subsequent generation of variant strains enables the virus to escape cellular and humoral immune responses that would normally control viremia. However HIV-infected cells are reported to secrete Tat in cell culture experiments [129] and in vivo [130,131]. Tat lacks a conventional signal sequence and is secreted through an unconventional secretion pathway utilized by fibroblast growth factor 2 [132], which requires the phospholipid component of the inner leaflet of the plasma membrane, phosphatidylinositol 4,5-bisphosphate (PtdIns(4,5)P2) [133]. The secreted Tat can be passed into uninfected cells, where it can render the cells more susceptible to infection [134], disrupt immune response by inducing apoptosis of T lymphocytes [135], and down-modulate expression of MHC class I [136]. In addition, the secreted Tat can traffic to the central nervous system by crossing the blood–brain barrier [137], where it plays a role in HIV-associated neurocognitive disorders [138,139]. While antibodies against Tat can block cellular uptake in a cell based in vitro assay system [140], anti-Tat antibodies are relatively uncommon in HIV^+^ people and have been shown to decline with time [141]. Nevertheless, reports that the presence of anti-Tat antibodies in serum of HIV^+^ people correlates with decreased progression to AIDS [142,143,144,145] support the need for research into a safe and effective vaccine that elicits cellular and humoral immune responses to Tat.

**Figure 2:**
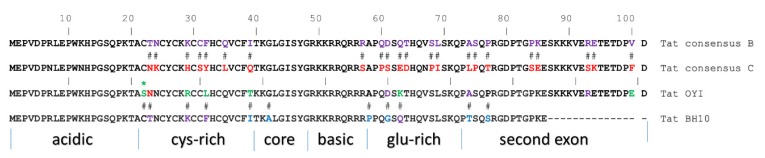
(a) Amino acid alignments for showing a consensus sequence for subtypes B and C [150], compared to the sequences of Tat-Oyi and Tat-BH10. Residues found in the subtype B consensus sequence (purple) and subtype C consensus (red) are indicated in the sequences of Tat-Oyi and Tat-BH10. Residues found in the consensus sequences of Tat-Oyi (green) and Tat-BH10 (blue) are also shown. The domains of Tat are demarcated.

HIV viruses are classified into four groups: M (major), O (outlier), N (non-M non-O) and P; a vast majority of HIV infections are induced by group M, which includes 9 subtypes and more than 70 circulating recombinant forms [146]. Vaccines undergoing animal and human clinical trials have used Tat protein derived from HIV strain BH10 [147] and Oyi [148], which are both group M subtype B viruses. There are differences in the primary amino acid sequences between Tat-BH10 and Tat-Oyi (Figure 2). Tat-BH10 is an 86 amino acid protein while Tat-Oyi, like a majority of Tat proteins in nature, has 101 amino acids. Tat-BH10 is a functional protein capable of transactivating the HIV LTR promoter, while Tat-Oyi is not. A Tat-Oyi C22S amino acid substitution, which is not present in Tat-BH10 and all other functional Tat isolates, can explain its defective phenotype in transactivation [149]. Finally, vaccination clinical trials have used Tat-BH10 purified from *E. coli*, while the Tat-Oyi used in vaccine trials was synthesized.

Both types of Tat vaccine were tested in non-human primate (SHIV) models, producing conflicting results regarding efficacy [80,81,87,151,152,153]. Here we recap recent small-scale phase l/lla vaccination clinical trials with Tat-BH10 or Tat-Oyi [78,79,82,86,154,155] (Table 1). In these trials, HIV-infected participants were treated with cART, which controlled viremia. The Tat-BH10 trial (see ISS T-002, also referred to as NCT0102455) did not include a double blinded, randomized controlled arm but did include a non-vaccinated HIV^+^ matched group for comparisons [84]. Vaccinees in the ISS T-002 study have been monitored for up to 8 years. A parallel Tat-BH10 trial (ISS T-003, yet to report) is randomized, blinded and includes a placebo arm. The Tat-Oyi trial was a randomized controlled study involving 48 people in four groups followed for 12 months [86]. Interestingly, optimal immune responses were reported when Tat-BH10 and Tat-Oyi were administered intradermally at 30 or 33 µg, respectively, and given 3 times, once monthly and without adjuvant. Both vaccines were found to be safe, and each vaccine produced humoral antibodies that recognize Tat. Tat-BH10 vaccination (ISS T-002) resulted in anti-Tat IgG, IgM and IgA antibodies being detected in ~90% of vaccinees in 6 months, which waned to ~37% after 8 years [83]. It is reported that only 12% to 20% of HIV^+^ people with chronic infection have antibodies to Tat [141,144,156,157,158,159]. At the start of the Tat-Oyi trial, >65% of subjects had no or low levels of antibodies to Tat. From months 5 to 12 post-inoculation with 33 µg or 96 µg of Tat-Oyi, ~70 to 80% of Tat-Oyi vaccinees developed anti-Tat IgG antibodies that recognize Tat from two to six of the most common HIV subtypes. However, these responders were not tested for levels of anti-Tat IgM and IgA. The number of responders with anti-Tat antibodies to at least three subtypes diminished by about 33% after 12 months post-inoculation compared to pre-inoculation levels. Likewise, Tat-BH10 vaccine responders produced antibodies that recognize Tat for HIV subtypes A, B, C and D. These antibodies may be therapeutic. Tat-BH10 responders had increased levels of CD4^+^ cells compared to baseline, as well as reduced viral loads in plasma and proviral DNA load in PBMCs [79,83]; and Tat-Oyi vaccine responders had reduced viral rebound following cessation of cART [86]. The evidence suggests that restoration of cell mediated immunity contributed to decreased proviral load. Clearly, these are encouraging outcomes that warrant further investigations.

Some questions remain regarding the overall efficacy of vaccines based on a single Tat protein. Although Tat functional domains from human and primate lentiviruses are relatively well conserved [160], numerous amino acid substitutions have been identified throughout the length of Tat [161]. As Tat requires functional domains to interact with cellular factors and TAR RNA for transactivation of HIV transcription, viral escape through Tat adaptation to an immune response could be limited. An early study using Tat peptides identified the Tat cysteine-rich and basic domains as epitopes for IgM responses to Tat [162], but there is evidence that Tat, although a poorly structured, unfolded protein [163], most likely presents conformational epitopes [85]. Interestingly an analysis of more than 300 Chinese HIV^+^ people with Tat antibodies showed neutralizing potential that correlated with a Tat N-terminal bias (amino acids 1–48) [157], although it is not known if anti-Tat responses in the Chinese cohort delayed disease progression. Finally, a new vaccine against Tat must be effective in a populations where there will be diverse HLA class-I and class-II variations, which may influence the overall efficacy of a Tat-based vaccine.

## 8. Conclusions

cART has greatly improved quality of life and decreased progression to AIDS for HIV^+^ people. The advent of pre-exposure prophylaxis (PrEP) [164], where uninfected people at high risk of contracting HIV infection take cART, has reduced new infections. While viral loads in HIV^+^ people can be strongly suppressed by cART, successful treatment for longer than 10 years does not appreciably decrease the size of the latent reservoirs [165], and for this reason interruption of cART results in a rapid viral rebound. HIV latency is established in resting T cells where little or no P-TEFb can be measured [166,167]. We suggest that the development of an agent that inhibits newly synthesized Tat and therefore the production of virus by infected cells would be an important step towards a functional cure [127]. Both Nullbasic and HT1 can strongly inhibit HIV transcription, and the former has been tested in mouse models of acute HIV infection. Their application would most likely require a gene therapy approach in hematopoietic stem cells. Whether immune cells protected from HIV infection by Nullbasic or HT1 would escape preexisting humoral and cellular immune responses to Tat is unknown. However, a phase I/II clinical trial of a TDN Rev called RevM10 showed stable expression for >100 weeks and preferential survival of transduced cells in one patient [168]. dCA has the remarkable ability to inactivate Tat and pre-clinical studies suggest that it can “lock” the HIV promoter so that transcription by RNAP II is inhibited durably even after dCA treatment is stopped. However, large-scale dCA production may be difficult [100], which could limit its availability. As the molecular interaction between dCA and Tat has been modeled [74], a future in silico screening project using pharmacophore based models of dCA interaction with Tat may identify additional agents with anti-Tat activity. Drug repurposing of the rheumatoid arthritis drug triptolide as an HIV inhibitor awaits safety and efficacy outcomes in clinical trials. Triptolide affects global transcription, which may limit its utility in safely controlling HIV infection. Anti-Tat vaccines have advanced to small clinical trials and 8-year follow-up results of a TatBH10 vaccine appear promising. Successive trials will be required to test the efficacy in a large cohort of HIV^+^ people. As no anti-Tat agents are available for clinical use, additional translational research of anti-Tat strategies should be a priority task.

## Figures and Tables

**Table 1:** Anti-Tat agent summary.

Anti-Tat Agent	Type	Inhibits; Proposed Mechanism(s)	Reference(s)
Nullbasic	Mutant Tat protein	1.Tat transactivation; binds to P-TEFb	[64,68,69,70]
		2. Reverse transcription; binds to reverse transcriptase	[64,65]
		3. Rev; binds to DDX1	[64,66,67]
HT1	HEXIM1-Tat fusion	Tat transactivation; binds to P-TEFb and inactivates CDK9	[71,72]
dCA	Small compound	1. Tat transactivation; binds Tat basic domain and prevents interaction with TAR RNA	[46,73,74]
		2. HIV transcription levels; heterochromatin formation on HIV LTR promoter	[75,76]
Triptolide	Small compound	1. Decreases Tat steady state levels; mechanism unclear	[77]
Tat-BH10	Vaccine	1. Tat; Tat neutralizing antibodies	[78,79]
		2. Virus production; anti-Tat cellular responses	[78,80,81]
		3. Provirus DNA load in blood lymphocytes; anti-Tat humoral/cellular responses	[82,83]
		4. Reduced immune activation; possibly due to reduced viral burden	[82,84]
Tat-Oyi	Vaccine	1. Tat; Tat neutralizing antibodies	[85,86]
		2. Virus production, mechanism unclear	[86]
		3. Viral rebound after treatment interruption; mechanism unclear	[86]
		3. Provirus DNA; mechanism unclear	[86,87]
